# Use of Fertigation and Municipal Solid Waste Compost for Greenhouse Pepper Cultivation

**DOI:** 10.1100/2012/973193

**Published:** 2012-05-03

**Authors:** Nikos Tzortzakis, Sofia Gouma, Eleni Dagianta, Christos Saridakis, Maria Papamichalaki, Dimitrios Goumas, Thrassyvoulos Manios

**Affiliations:** ^1^Department of Organic Greenhouse Crops and Floriculture, School of Agricultural Technology, Technological Educational Institute of Crete, 71004 Heraklion, Greece; ^2^Department of Crop Science, School of Agricultural Technology, Technological Educational Institute of Crete, 71004 Heraklion, Greece

## Abstract

Municipal solid waste compost (MSWC) and/or fertigation used in greenhouse pepper (*Capsicum annuum* L.) cultivation with five different substrates with soil (S) and/or MSWC mixtures (0–5–10–20–40%) used with or without fertigation. Plants growth increased in 10–20% MSWC and fertigation enhanced mainly the plant height. Fruit number increased in S : MSWC 80 : 20 without fertilizer. Plant biomass increased as MSWC content increased. There were no differences regarding leaf fluoresces and plant yield. The addition of MSWC increased nutritive value (N, K, P, organic matter) of the substrate resulting in increased EC. Fruit fresh weight decreased (up to 31%) as plants grown in higher MSWC content. Fruit size fluctuated when different MSWC content used into the soil and the effects were mainly in fruit diameter rather than in fruit length. Interestingly, the scale of marketable fruits reduced as MSWC content increased into the substrate but addition of fertilizer reversed this trend and maintained the fruit marketability. MSWC affected quality parameters and reduced fruit acidity, total phenols but increased fruit lightness. No differences observed in fruit dry matter content, fruit firmness, green colour, total soluble sugars and EC of peppers and bacteria (total coliform and *E. coli*) units. Low content of MSWC improved plant growth and maintained fruit fresh weight for greenhouse pepper without affecting plant yield, while fertigation acted beneficially.

## 1. Introduction

Over 500 kg of municipal solid waste (MSW) per inhabitant and year are generated in European Union [1]. Worldwide residue generation has increased considerably over the last 30 years, entailing not only the loss of materials and energy, but also negative environmental impacts. Many studies have shown that the application of immature composts to soil causes severe damage to plant growth [2]. Composting could turn large volumes of MSW into material to be used as fertilizer, organic soil additive and crop substrate. There are, however, certain limitations on some composts use with marked variation in physical/chemical properties (i.e., porosity, and salt content).

Municipal solid waste compost (MSWC) as an organic soil additive when applied in field trials suggested that it can be used in agricultural production, improving soil physicochemical properties, increasing water retention as well as supply with considerable amount of essential nutrients [3, 4]. Numerous studies have addressed the use of compost in nursery plant production, and have analyzed its chemical, physical, and biological properties [5, 6]. It has been found that mixtures of compost with perlite (20–50% MSWC) in nurseries may be used as substrates without the need for additional mineral fertilizer [7]. Maynard [8] reported 58% higher yield in tomato crop amended with 11.2 t ha^−1^ MSW compost but noticed symptoms of damping off diseases and dying in squash. Ozores-Hampton et al. [9] reported improved tomato growth and yield after applying MSW compost but questioned the high cost compared to commercial fertilizer. However, little information is available regarding the use of MSWC as an additive into the soil in horticultural crops as well as in impacts of fruit quality.

Fruit quality encompasses many aspects and includes not only flavour, colour, nutritional aspects, and firmness, but also shelf life, processing attributes, and resistance to pathogens [10]. At the market interface, only product tshat corresponds to the expectations of the consumer can survive. Fruit firmness is an important quality attribute and is directly related to enhance the storability potential and to induce greater resistance to decay and mechanical damage [11]. Moreover, an increased interest in vegetables has been created by the fact that their consumption has been correlated to the human health and the reduced risk of some types of cancer [12].

The present study sought to evaluate the impacts of mineral fertigation combined with different content of MSWC mixed with soil, in plant growth, and fruit quality-related parameters in greenhouse pepper production.

## 2. Material and Methods

### 2.1. Plant Material and Municipal Solid Waste Compost Source

Pepper (*Capsicum annuum* L. cv. Oregon) plants were grown under natural light from September to January (in 2010-2011). Municipal solid waste compost (MSWC) punctuated by Inter-Municipal Enterprise for the Management of Solid Wastes (IMEMSW), based in Chania. The compost used was made from the organic fraction of selectively collected urban waste. The composting procedure lasted for 5-6 months. The 60% of compost consisted of particles with <4 mm size.

### 2.2. Experimental Design

The experiment was carried out in an unheated plastic greenhouse with a North-South orientation at the Technological Educational Institute of Crete, Greece. Seedlings were produced in plastic seedling trays filled with expanded clay and were acquired from local agriculture nursery. Two medium, soil (S), and municipal solid waste compost (MSWC), and mixtures of these, were used to create five substrates which were (1) S : MSWC (100 : 0) as control, (2) S : MSWC (95 : 5), (3) S : MSWC (90 : 10), (4) S : MSWC (80 : 20), and (5) S : MSWC (60 : 40). The substrates were irrigated with water and/or fertilizers which resulted in 10 treatments (3 replication/treatment; 3 plants/replication).

Seedlings were transplanted in single pots (filled with substrate; 9 L capacity pot) and arranged in a single row with a completed randomized design for the replicates/treatment. Rows were 1.0 m apart, and plants were separated by 0.4 m. Drip irrigation emitters (1 emitter/pot) were placed and irrigation took place twice (1 min/time) daily, through timer, by means of pressure pumps. Fertigation (EC: 2.5–3.0 mS/cm; 200 mL/plant twice a week) with commercial fertilizers took place manually. The drainage solution was collected in trays in each pot and was available for plant water needs through capillary suction.

### 2.3. Measurements

Physicochemical properties of substrates were observed. Thus, it was measured K and Na content (by a flame photometer), P (spectrophotometrically), total N (Kjeldahl), organic matter content and organic carbon, pH, and EC.

Beginning the second week after transplanting, it was studied the impact of substrate medium on plant growth/development and yield in pepper. It was measured every second week the plant height, main stem diameter, leaf number produced, flower and fruit number, and the leaf fluoresces. With the completion of the experiment, plant biomass (fresh weight and the % of dry matter) and plant yield were determined.

Fruit fresh weight, dry matter content (%), and fruit size (length and diameter) were measured. Fruit marketability observed by employing a 1–4 scale (1: extra quality; 2: good quality; 3: medium quality (i.e., small size, decolourization); 4: not marketable quality (i.e. malformation, wounds, and infection). Fruit colour measurements taken around the fruit equator (2 measurements per fruit) with a Minolta Chroma Meter CR300. Data are expressed in L × a × b units. Fruit firmness was measured at 1 point on the shoulder of the fruit, for each treatment, using a penetrometer FT 011 (TR Scientific Instruments, Forli, Italy). The amount of force (in Newtons; N) required to break the radial pericarp (i.e. surface) of each tomato was recorded at ambient (22–24°C) temperature.

Total soluble solids (TSSs) concentration was determined on the fruit juice for each treatment with a digital refractometer 300017 (Sper Scientific Ltd, AZ, USA) at 20°C and results were expressed as the mean (%) of °Brix. Subsamples of homogenised fruit tissue were used to determine the pH of fruit juice using a standard pH meter (Orion 920A, Scientific Support, Hayward, CA, USA). Titratable acidity (TA) was determined by potentiometric titration, using fruit samples (5 g) diluted in 100 mL distilled water and titrated with 0.1 N NaOH, using phenolphthalein as pH indicator, and monitored up to 8.2 end point with a pH-meter. The reported values were expressed in terms of citric acid percentage. Total phenolics was determined on blended fruit tissue (5 g) extracts following repeated (4-fold) addition of 2.5 mL of 50% (v/v) methanol as reported previously [13]. Results were expressed in terms of gallic acid equivalents (GAE; Sigma Aldrich, Athens, Greece) per 100 g fresh weight of tissue.

Fruits were assessed for bacteria (total coliform and *Escherichia coli*) units on the fruits as well as in the fruit, by employing Chromocult *Coliform Agar *(Merck KGaA).

### 2.4. Statistical Analysis

Data were tested for normality, and then subjected to analysis of variance (ANOVA). Significant differences between mean values were determined using Duncan's Multiple Range test (*P* < 0.05) following one-way ANOVA. Significant differences on percentage values (% dry matter) were logarithmatically transformed prior to analysis. Statistical analyses were performed using SPSS (SPSS Inc., Chicago, Ill.) and graphs produced using Prism v.2.0 (Graph Pad Inc., San Diego. Calif.).

## 3. Results and Discussion

### 3.1. Substrate Properties

The elemental analysis of the pure MSWC revealed pH: 7.52; EC: 16.54 mS/cm; organic C: 26.62%; total N: 0.57%; P: 164 ppm; K: 727 ppm; Na: 403 ppm. Thus the addition of MSWC, as organic medium, into the soil increased the organic matter content (as a consequence the organic C content) and the pH and EC of the medium (Table 1), and the values are in agreement with previous studies [14]. Increasing the amount of MSWC into the soil resulted in increased N, P, K, and Na content which alters the nutritive value of the medium. Municipal solid waste is approximately 60–90% biodegradable and might be used as a bulking material to absorb excess water and supply a useful raw product for the horticulture industry [15]. After application of waste, an increase in the electrical conductivity of soil has also been reported in other studies (as reviewed by Asgharipour and Armin [16]). The increase in nutrient concentrations in soil solution after using compost reduces the activity of soil microorganisms [17] and affects the micronutrient absorption by plant [18].

### 3.2. Effect on Plant Growth

**Figure 1:**
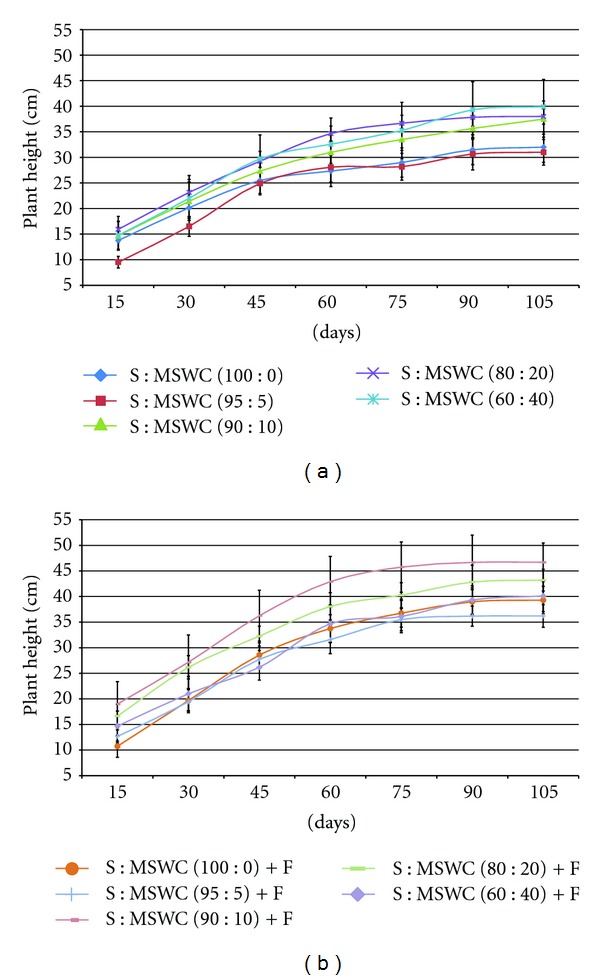
Effects of municipal solid waste compost (MSWC) into soil (S) without (a) or with (b) fertigation (F) on plant height of greenhouse pepper crop. Values represent mean (±SE) of measurements made on six plants per treatment.

**Figure 2:**
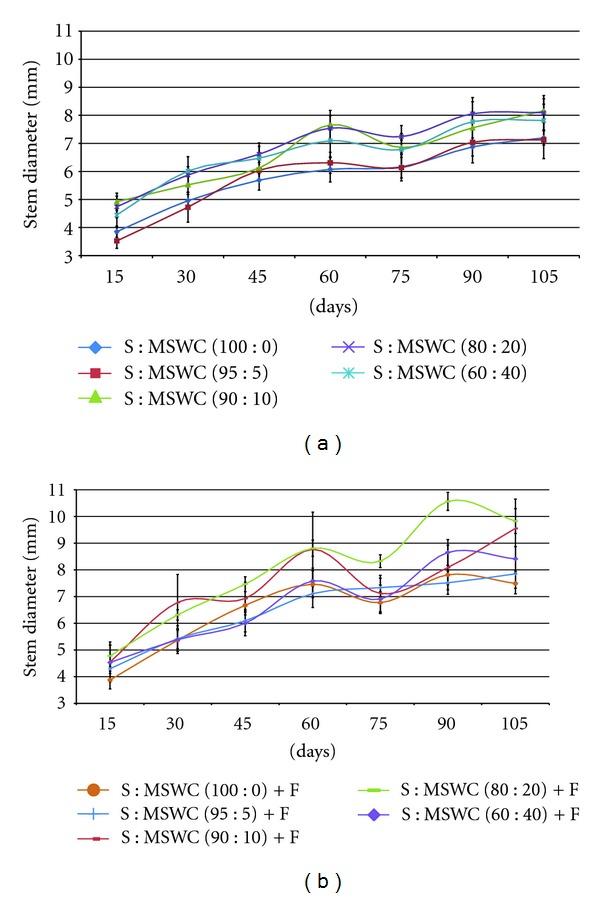
Effects of municipal solid waste compost (MSWC) into soil (S) without (a) or with (b) fertigation (F) on stem diameter of greenhouse pepper crop. Values represent mean (±SE) of measurements made on six plants per treatment.

**Figure 3:**
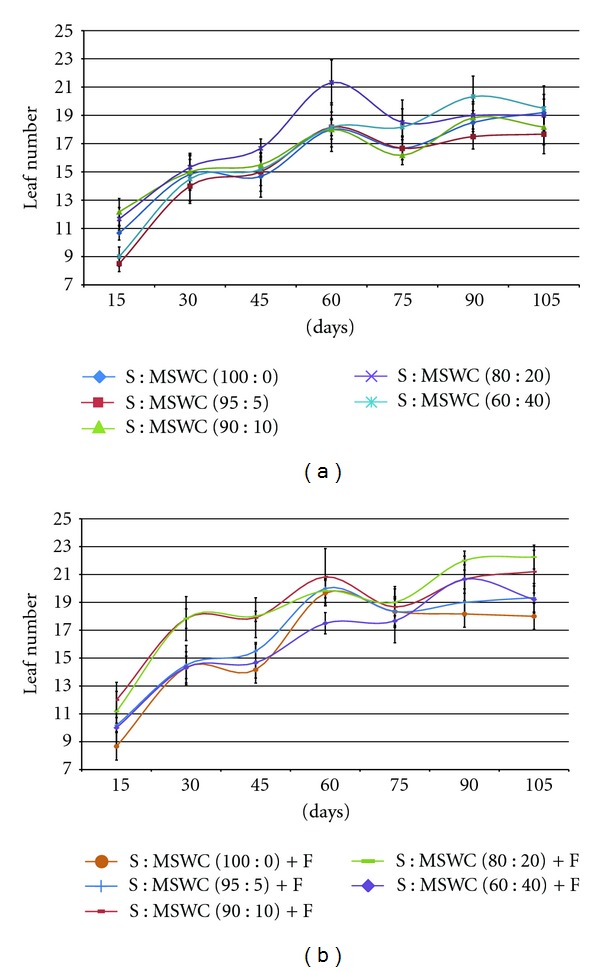
Effects of municipal solid waste compost (MSWC) into soil (S) without (a) or with (b) fertigation (F) on leaf number produced in greenhouse pepper crop. Values represent mean (±SE) of measurements made on six plants per treatment.

Examining the different MSWC content into the soil, plants grown in mixtures with MSWC ≥10% were taller as well as revealed thicker (stem diameter) main stem (up to 13%) comparing with the control (S) following 105 days growth (Figures 1(a) and 2(a)). The addition of fertilizers improved plant height and stem thickness only in case of S : MSWC 90 : 10 and S : MSWC 80 : 20 (Figures 1(b) and 2(b)), while greater MSWC content (i.e. 40%) with fertigation did not differ with the control treatment. Similar effects were observed in potted geranium plants in various MSWC content [19]. There were no differences in leaf number produced in plants grown in different MSWC content comparing with the control, while, the fertigation add enhanced leaf number in plants grown in S : MSWC 90 : 10 and S : MSWC 80 : 20 (Figures 3(a) and 3(b)).

**Figure 4:**
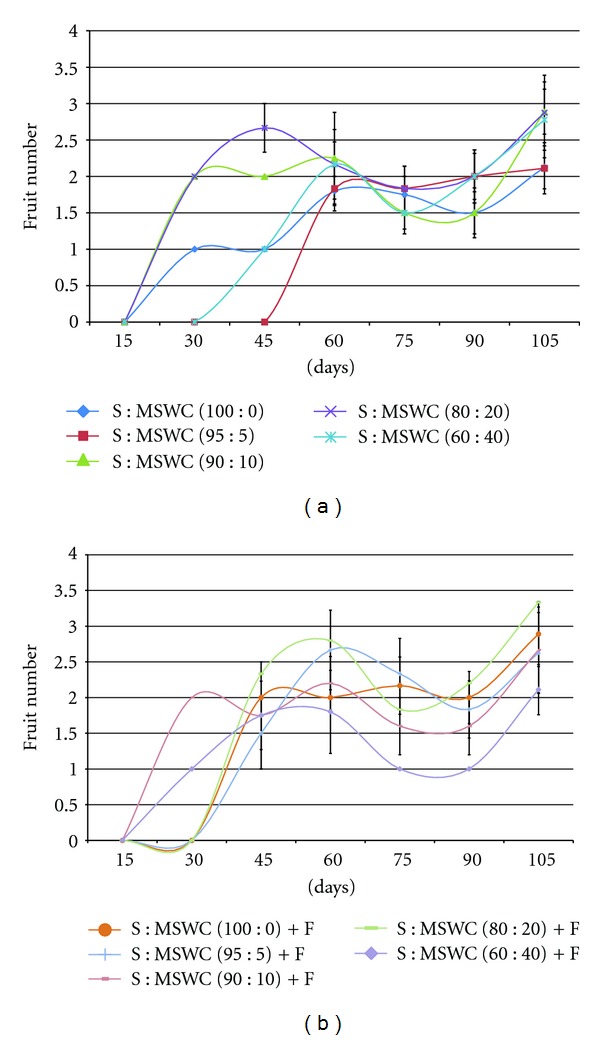
Effects of municipal solid waste compost (MSWC) into soil (S) without (a) or with (b) fertigation (F) on fruit number produced in greenhouse pepper crop. Values represent mean (±SE) of measurements made on six plants per treatment.

Fruit number produced followed similar pattern with the number of flower produced (data not presented). At the begging (the first 45 days) of the experiment, fruit number was doubled in plants grown in S : MSWC 90 : 10 and S : MSWC 80 : 20 comparing with the control, while in the end, S : MSWC 80 : 20 mixture revealed the greatest fruit number (Figure 4(a)). No differences were observed in leaf fluoresce among MSWC content with or without the addition or fertilizers (data not presented).

### 3.3. Effect on Plant Yield

**Figure 5:**
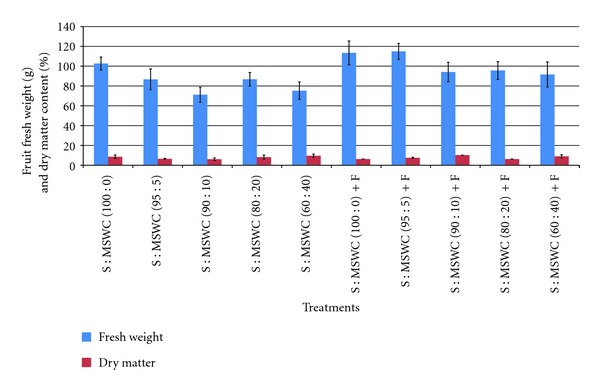
Effects of municipal solid waste compost (MSWC) into soil (S) without or with fertigation (F) on fruit fresh weight (g) and dry matter content (%) of greenhouse pepper crop. Values represent mean (±SE) of measurements made on average 23 fruits per treatment.

Fruit fresh weight significant reduced in plant grown in S : MSWC 90 : 10 and S : MSWC 80 : 20 comparing with the control (Figure 5) while no differences were observed in plant yield (Table 2), and this is due to the increased fruit number (i.e. more fruits) (see Figure 4). For the same substrates, when fertilizers added, fruit weight reduced. Interesting, fertigation in S : MSWC 60 : 40 reduced plant yield while no differences were observed in less MSWC content and/or control treatment (Table 2). At 40% MSWC, the yield decrease is a likely result of salt stress and would promote the maximum EC tolerable by this plant. In previous studies, when geranium plant was grown in 20% of MSWC, the maximum EC tolerable by this plant achieved [20], which differ with the present results, as pepper is probably a more competitive crop in nutrients and/or salt resistance than geranium crop. Therefore, the MSWC rates must be adjusted according to the conductivity of the applied compost and to the salt tolerance characteristic of the plant species to avoid salt stress and detrimental effects on plant growth. As MSWC content increased into the substrate, plant biomass fresh weight increased while biomass dry matter reduced (Table 2).

Roe [20] reported a higher zinc concentration in soil and a low germination rate of squash after application of MSW compost. Maynard [8] reported 58% higher yield in tomato crop amended with 11.2 t ha^−1^ MSW compost but noticed symptoms of damping-off diseases and dying in squash. Ozores-Hampton et al. [9] reported improved tomato growth and yield after applying MSW compost but questioned the high cost compared to commercial fertilizer.

### 3.4. Effect on Fruit Quality

**Figure 6:**
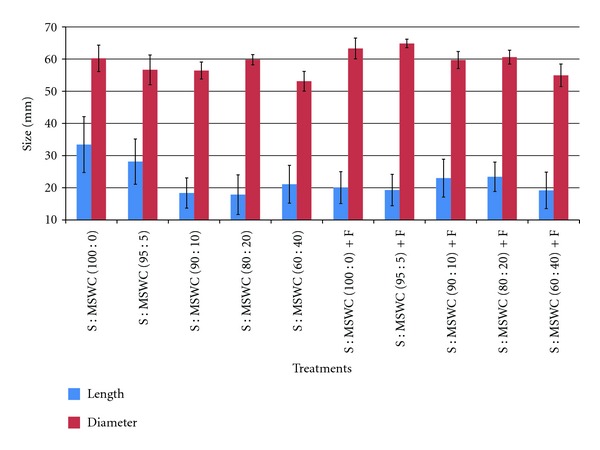
Effects of municipal solid waste compost (MSWC) into soil (S) without or with fertigation (F) on fruit size (length and diameter in mm) of greenhouse pepper crop. Values represent mean (±SE) of measurements made on average 23 fruits per treatment.

Regarding fruit quality-related parameters, fruit fresh weight reduced in substrates containing MSWC greater than 5% (independently of the fertigation) while no differences were observed in fruit dry matter content (Figure 5). Fruit size fluctuated when different MSWC content was used into the soil and the effects were mainly in fruit diameter (i.e. thicker fruits) rather than in fruit length (Figure 6). In details, fruit length reduced in case of the substrate S : MSWC 60 : 40 comparing with the control in both fertigation and nonfertigation application. However, fruit diameter reduced when MSWC content into the soil was greater than 5% while fertigation alleviated this reduction and fruit diameter maintained among treatments. However, the dry matter and the visual health of potatoes were significantly improved when fertilizer applied compared to those received MSWC [21], which differ with the present study, and this is possible due to the different crop and/or different MSWC and fertilizer application. 

**Figure 7:**
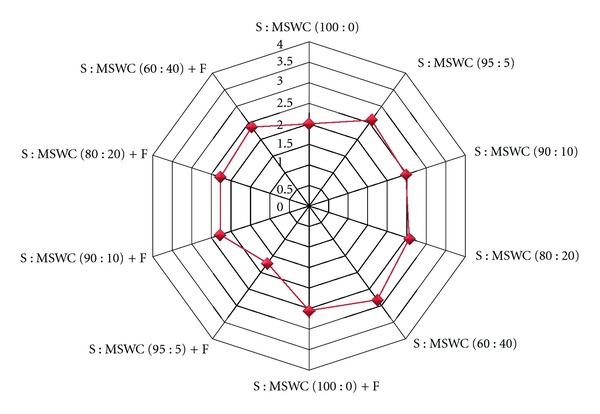
Effects of municipal solid waste compost (MSWC) into soil (S) without or with fertigation (F) on fruit marketability in 1–4 scale (1: extra quality; 2: good quality; 3: medium quality; 4: not marketable quality) of greenhouse pepper crop. Values represent mean of measurements made on average 23 fruits per treatment.

Fruit marketability was maintained as good quality (marked with 2 out of 4 values) for the control treatments and medium quality (marked with 2.5 out of 4 values) in case of MSWC used into the soil (Figure 7). When fertigation applied, fruit marketability was slightly improved, and this is due to the better nutrition that plants achieved. 

Fruit lightness (L) increased when MSWC content was greater than 10% into the substrate while the addition of fertilizer increased L value only when MSWC content was greater than 40% (Table 3). Fruit green colour (chroma a and b) fluctuated in different MSWC content with/without fertilizer. No difference was observed in fruit firmness among treatments (Table 3).

Peppers were less acid in plants grown in 10–20% MSWC content as the pH fruit juice increased but no differences were observed in the pepper juice EC. Acidity, expressed in citric acid percentage, reduced in case of S : MSWC 60 : 40 substrate comparing with the control while the addition of fertilizer increased acidity of fruits harvested in plants grown in S : MSWC 90 : 10 and S : MSWC 80 : 20 substrates. No differences observed in total soluble sugars of pepper juice among treatments which resulted in no differences in fruit sweetness (Table 4), which is in accordance with previous studies when MSWC applied in strawberry crop [22]. Total phenols content decreased (up to 46%) with the addition of MSWC into the substrate while fertilizer addition enhanced the phenols reduction.

Bacteria (total coliform and *E. coli*) units on the fruits did not differ among the treatments. In details, the average Total Coliform units were 5.3 CFU/100 g fwt while fertigation enhanced the bacteria presence. The average number of *E. coli *was approximately 0.36 CFU/100 g fwt, and it was only presence in case of the application of fertilizer in S : MSWC (100 : 0) and S : MSWC (90 : 10) substrates. When selected fruit examined for the bacteria presence inside the fruit, bacteria units were not detected, by the meaning that bacteria probably did not move through plant tissue. However, this fact needs to be examined more precisely and details in future, before final statements. It's worthwhile to mention that the bacteria loan into the soil was approximately 10^3^ times more than the one in fruits, for the equivalent treatments.

## 4. Conclusions

The production of MSWC is an important recycling opportunity for many communities; however, the safety of its use in agriculture has been debated because of concerns over the levels of its salt and metals content [23]. Salinity seems to be the major limiting factor to the use of large amounts of MSWC as a growth-media component. Therefore, the MSWC rates must be adjusted according to the conductivity of the applied compost and to the salt tolerance characteristic of the plant species to avoid salt stress and detrimental effects on plant growth. Thus, the addition of fertilizer into the substrate alleviated the negative impacts of the increased MSWC content and maintained fruit fresh weight. MSWC content among 10–20% into the substrate may benefit plant growth and fresh fruit weight, especially when fertigation take place.

## Figures and Tables

**Table 1:** Physicochemical properties of substrate medium consisted of soil (S) and municipal solid waste compost (MSWC) resulted in five medium.

	Organic matter (%)	Organic C (%)	pH	EC (mS/cm)	Total N (%)	C/N	P (ppm)	K (ppm)	Na (ppm)
S : MSWC (100 : 0)	0.825	0.48	6.94	0.71	0.014	21.7	21.73	5.55	0.32
S : MSWC (95 : 5)	1.513	0.88	7.17	1.38	0.024	31.9	31.97	11.38	10.24
S : MSWC (90 : 10)	2.098	1.22	7.33	2.03	0.056	53.8	53.79	25.93	26.11
S : MSWC (80 : 20)	2.304	1.34	7.51	3.39	0.081	58.4	58.45	43.40	40.99
S : MSWC (60 : 40)	4.506	2.61	7.58	7.35	0.168	79.8	79.88	124.37	117.02

**Table 2:** Effects of municipal solid waste compost (MSWC) into soil (S) with or without fertigation on yield (g/plant), upper biomass fresh fruit (g/plant), and upper biomass dry matter (%) in greenhouse pepper crop.

	Water			Fertigation		
	Yield (g/plant)	Biomass (g/plant)	Biomass dry content (%)	Yield (g/plant)	Biomass (g/plant)	Biomass dry content (%)
S : MSWC (100 : 0)	221.75 a^Y^	57.86 b	47.12 a	327.61 a	68.61 c	41.71 a
S : MSWC (95 : 5)	183.04 a	74.95 ab	37.85 b	301.72 ab	75.01 bc	38.52 ab
S : MSWC (90 : 10)	205.33 a	77.63 a	38.82 b	250.78 ab	95.51 ab	37.37 ab
S : MSWC (80 : 20)	205.98 a	75.13 a	40.35 b	318.72 a	100.76 a	34.15 b
S : MSWC (60 : 40)	208.97 a	84.43 a	38.48 b	193.37 b	86.08 b	36.67 b

^Y^Values (*n* = 6) in columns followed by the same letter are not significantly different. *P* ≤ 0.05.

**Table 3:** Effects of municipal solid waste compost (MSWC) into soil (S) with or without fertigation on fruit firmness (N) and fruit color (L, a, and b values) in greenhouse pepper crop.

		Fruit firmness	Fruit color		
			L	a	b
No fertilizer	S : MSWC (100 : 0)	4.40 a^Y^	33.69 b	−10.81 ab	14.37 b
	S : MSWC (95 : 5)	3.46 b	33.55 b	−10.52 a	13.28 b
	S : MSWC (90 : 10)	4.33 ab	35.40 a	−12.65 c	17.36 a
	S : MSWC (80 : 20)	4.26 a	35.16 a	−11.34 b	15.02 b
	S : MSWC (60 : 40)	3.96 ab	36.28 a	−11.48 bc	16.10 ab

With fertilizer	S : MSWC (100 : 0)	4.40 a	34.16 b	−10.57 b	13.62 ab
	S : MSWC (95 : 5)	3.41 a	33.59 b	−8.84 a	10.93 c
	S : MSWC (90 : 10)	3.15 a	34.98 ab	−11.76 c	15.61 a
	S : MSWC (80 : 20)	3.73 a	34.24 b	−9.50 ab	12.10 b
	S : MSWC (60 : 40)	3.23 a	36.35 a	−12.43 c	16.94 a

^Y^Values (*n* = 9) in columns followed by the same letter are not significantly different. *P* ≤ 0.05.

**Table 4:** Effects of municipal solid waste compost (MSWC) into soil (S) with or without fertigation on total soluble solids (TSS: °Brix), titratable acidity (TA: (% citric acid), sweetness (TSS/TA), Ph, EC (mS/cm), and total phenols (gallic acid equivalent: GAE/100 g fwt)) in greenhouse pepper crop.

		TSS	TA	TSS/TA	pH	EC	Total phenols
No fertilizer	S : MSWC (100 : 0)	3.26 a^Y^	4.68 ab	0.79 ab	5.30 c	1.78 ab	296.91 a
	S : MSWC (95 : 5)	2.76 a	3.93 b	0.69 b	5.52 b	1.87 ab	176.56 b
	S : MSWC (90 : 10)	3.36 a	5.41 a	0.61 b	5.85 a	1.25 b	159.03 b
	S : MSWC (80 : 20)	2.53 a	4.04 b	0.64 b	5.39 bc	2.47 a	221.73 b
	S : MSWC (60 : 40)	3.20 a	3.04 c	1.04 a	5.41 bc	1.77 ab	176.76 b

With fertilizer	S : MSWC (100 : 0)	3.20 ab	3.09 c	1.13 a	5.30 c	1.48 a	184.57 a
	S : MSWC (95 : 5)	3.40 a	3.58 bc	0.95 a	5.93 b	1.57 a	125.94 c
	S : MSWC (90 : 10)	3.43 a	3.53 b	0.54 b	6.31 a	1.88 a	140.06 bc
	S : MSWC (80 : 20)	3.36 a	3.10 a	1.23 a	5.30 b	1.29 a	162.79 b
	S : MSWC (60 : 40)	2.73 b	3.82 bc	0.70 b	5.75 b	1.33 a	155.82 b

^Y^Values (*n* = 6) in columns followed by the same letter are not significantly different. *P* ≤ 0.05.
